# GM3 Ganglioside Linked to Neurofibrillary Pathology in a Transgenic Rat Model for Tauopathy

**DOI:** 10.3390/ijms222212581

**Published:** 2021-11-22

**Authors:** Dominika Olešová, Petra Majerová, Roman Hájek, Juraj Piešťanský, Radana Brumarová, Alena Michalicová, Bernadeta Jurkanin, David Friedecký, Andrej Kováč

**Affiliations:** 1 Institute of Neuroimmunology, Slovak Academy of Sciences, Dúbravská cesta 9, 84510 Bratislava, Slovakia; dominika.olesova@savba.sk (D.O.); petra.majerova@savba.sk (P.M.); alena.michalicova@savba.sk (A.M.); bernadeta.valachova@savba.sk (B.J.); 2 Laboratory of Biomedical Microbiology and Immunology, University of Veterinary Medicine and Pharmacy, 04181 Kosice, Slovakia; 3 Waters Corporation, Stamford Avenue, Altrincham Road, Wilmslow SK9 4AX, UK; roman_hajek@waters.com; 4 Department of Pharmaceutical Analysis and Nuclear Pharmacy, Faculty of Pharmacy, Comenius University in Bratislava, Odbojarov 10, 83232 Bratislava, Slovakia; piestansky@fpharm.uniba.sk; 5 Laboratory for Inherited Metabolic Disorders, Department of Clinical Biochemistry, University Hospital Olomouc, and Faculty of Medicine and Dentistry, Palacký University Olomouc, I. P. Pavlova 6, 77900 Olomouc, Czech Republic; radana.karlikova@gmail.com (R.B.); david.friedecky@upol.cz (D.F.)

**Keywords:** glycosphingolipids, gangliosides, sulfatides, tauopathy, neurodegeneration, aging, liquid chromatography, mass spectrometry

## Abstract

Glycosphingolipids (GSLs) are amphipathic lipids composed of a sphingoid base and a fatty acyl attached to a saccharide moiety. GSLs play an important role in signal transduction, directing proteins within the membrane, cell recognition, and modulation of cell adhesion. Gangliosides and sulfatides belong to a group of acidic GSLs, and numerous studies report their involvement in neurodevelopment, aging, and neurodegeneration. In this study, we used an approach based on hydrophilic interaction liquid chromatography (HILIC) coupled to high-resolution tandem mass spectrometry (HRMS/MS) to characterize the glycosphingolipid profile in rat brain tissue. Then, we screened characterized lipids aiming to identify changes in glycosphingolipid profiles in the normal aging process and tau pathology. Thorough screening of acidic glycosphingolipids in rat brain tissue revealed 117 ganglioside and 36 sulfatide species. Moreover, we found two ganglioside subclasses that were not previously characterized—GT1b-Ac2 and GQ1b-Ac2. The semi-targeted screening revealed significant changes in the levels of sulfatides and GM1a gangliosides during the aging process. In the transgenic SHR24 rat model for tauopathies, we found elevated levels of GM3 gangliosides which may indicate a higher rate of apoptotic processes.

## 1. Introduction

The central nervous system is known for its high lipid content and complexity. Still little is known about its precise role in cells. Glycosphingolipids (GSLs) are amphipathic lipids composed of a sphingoid base and a fatty acyl group attached to a mono-, or oligosaccharide moiety [1]. Within cells, GSLs are located mainly in the outer leaflet of the plasmatic membrane. The hydrophobic fatty acyl and sphingoid base are anchored in the membrane while the saccharide sticks out of the cell surface. Unlike phospholipids, GSLs are not homogeneously layered within the membrane, rather they are grouped with functional proteins and cholesterol in lipid rafts. Their main functions are modulation of signal transduction and directing proteins to specific places within the membrane [2]. Moreover, GSLs play an essential role in cell recognition and modulation of cell adhesion. The glycan part on the outer side of the membrane can be recognized not only by glycan-binding proteins and antibodies, but also bacteria and viruses [3].

Gangliosides (GSs) and sulfatides (STs) belong to a group of acidic GSLs and numerous studies report their involvement in neurodevelopment, aging, and neurodegeneration. GM2 ganglioside levels are elevated in neurons and microglia in ethanol-induced neurodegeneration [4]. Progressive accumulation of GSs is caused by lysosomal dysfunction and is followed by neurodegeneration [5]. Complement activation and neuroinflammation have been reported in ganglioside knock-out mice [6]. Aberrant sulfatide levels were detected in APP mice [7], multiple sclerosis [8], and Alzheimer’s disease [9]. However, there is no evidence of how neurofibrillary degeneration affects the metabolism of acidic GSLs. Moreover, the character of the ceramide moiety in GSLs, especially the sphingoid base, changes significantly during neurodevelopment, but also later during aging [10,11].

Tauopathies are a diverse group of neurodegenerative diseases characterized by abnormal accumulation of aggregated forms of tau protein in neurons and/or glial cells [12]. Tau proteins are microtubule-associated proteins that plays a pivotal role in the stabilization of microtubules of the cytoskeleton. Abnormal post-translational modifications of tau proteins lead to a pathological cascade of misfolding, aggregation, and subsequent accumulation in cells. Finally, depolymerization of microtubules causes impairment in axonal transport, synaptic dysfunction, and neurodegeneration [13]. Alzheimer’s disease is the most prominent tauopathy-associated neurological disease. Among more than 20 others are frontotemporal dementia with parkinsonism-17, progressive supranuclear palsy, corticobasal degeneration, chronic traumatic encephalopathy, and others [14].

In the presented work, we performed characterization of acidic glycosphingolipids in rat brain tissue and semi-targeted lipidomic screening in a transgenic rat model expressing human truncated tau proteins. We focused on the investigation of lipid changes in the normal aging process and tau pathology-induced neurodegeneration. This could lead to a better understanding of lipid pathophysiology in aging and neurodegeneration.

## 2. Results

### 2.1. Characterization of the Glycosphingolipid Profile in Rat Brain Tissue

Full scan negative ESI spectra were obtained from QC samples of brain tissue extracts and manually screened for known and possibly new glycosphingolipid species. This screening was based on a method published by Hajek et al. [15]. The identity of the newly discovered species was predicted based on chromatographic behavior and confirmed by fragmentation spectra. The elemental composition was determined according to accurate *m*/*z* measurements with an average mass accuracy of 1.68 ppm and the threshold for positive identification set to ±10 ppm. A total number of 117 ganglioside species belonging to 19 major subclasses and 36 sulfatide species were positively identified in rat brain tissue (Table S1).

**Figure 1:**
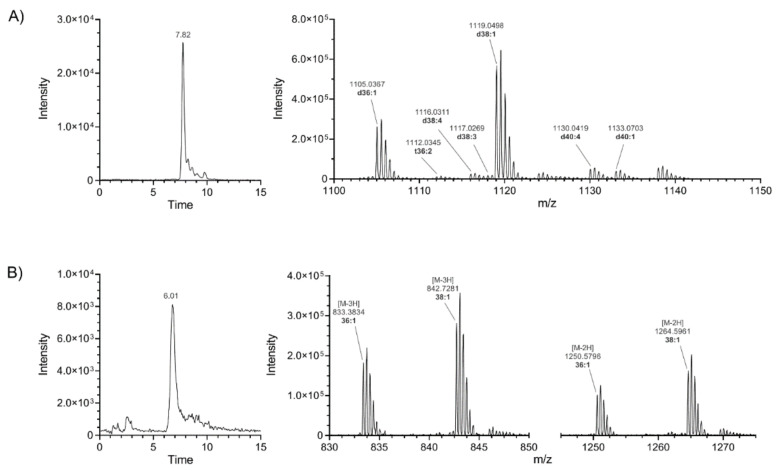
GT1b-Ac2 and GQ1b-Ac2 are newly characterized ganglioside subclasses in rat brain tissue. Representative RIC (reconstructed ion current) chromatograms (left) and HRMS spectra (right) of newly characterized ganglioside subclasses GT1b-Ac2 (**A**) and GQ1b-Ac2 (**B**).

**Figure 2:**
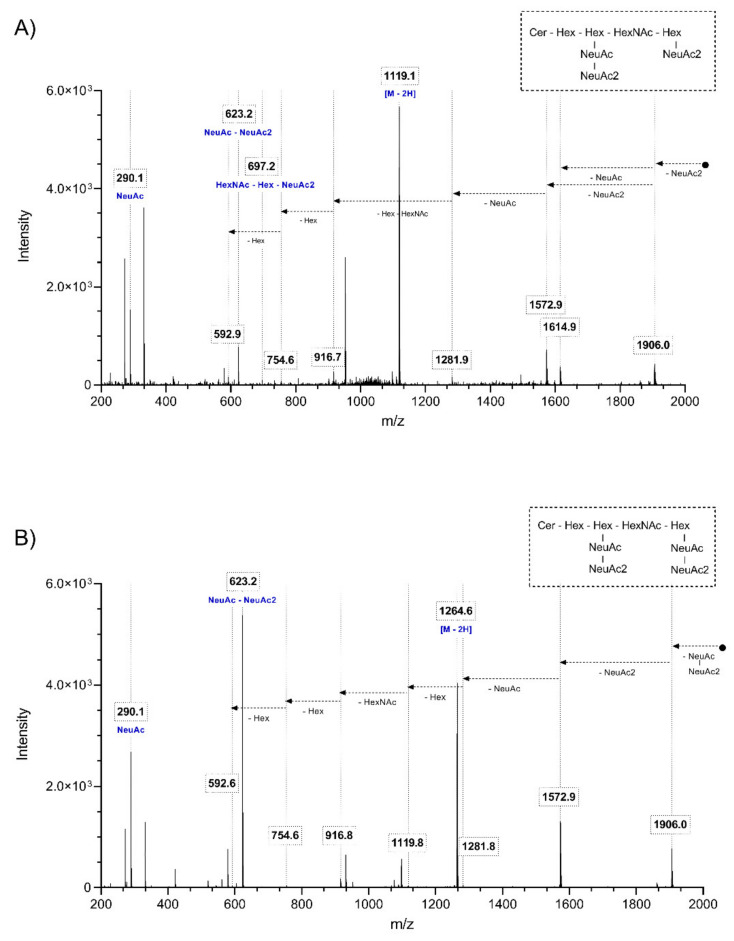
Fragmentation spectra of representative ganglioside species from both newly discovered ganglioside classes. LC-(ESI)-MS/MS spectra were obtained for the [M-H]^2−^ ion of GT1b-Ac2 at *m*/*z* 1119.1 (**A**), and the [M-3H]^3-^ ion of GQ1b-Ac2 at *m*/*z* 842.7 (**B**) in negative ion mode.

Two subclasses of gangliosides containing 11 species could not be found in previously published literature nor public lipid databases (SwissLipids, LipidMaps). Representative chromatograms and mass spectra for both subclasses are shown in Figure 1. Identification was carried out by inspecting the MSMS spectra and specific fragmentation patterns. These investigations revealed that unknown GS species belonged to subclasses GT1b-Ac2 and GQ1b-Ac2 (Figure 2). Theoretical *m*/*z* and elemental composition were determined by drawing the theoretical structure in silico. Subsequently, positive identification was obtained by comparing the theoretical and experimental *m*/*z*.

### 2.2. Glycosphingolipid Screening Revealed Changes in Ganglioside and Sulfatide Levels in Aging

First, we focused on lipid changes induced by the normal aging process. Data acquired from GSL quantification were processed and transformed for statistical analysis. First, unsupervised PCA was performed for survey group separation. No separation of 14-month-old TG vs. CN animals was present, however, slight separation was observed between young (CN4) and old (CN14) control animals (Figure S1A). Supervised PLS-DA was employed to verify the results from the unsupervised analysis (Figure S1B). Also, data were subjected to univariate analysis for significance testing. The results for all quantified lipids in all tested groups are summarized as a heatmap in Table S2.

**Figure 3:**
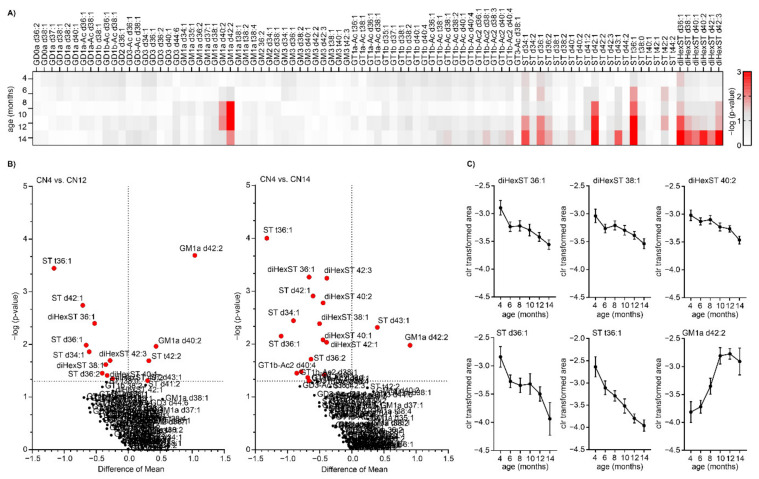
Altered levels of several ganglioside and ST species in the aging process. (**A**) Heatmap showing results from significance testing as -log10 (*p*-value). (**B**) Volcano plots showing the results from the statistical analysis of GSL profiles in 12-month-old and 14-month-old control animals compared with control animals (4-month-old). The dashed line represents a *p*-value threshold of 0.05. (**C**) Confidence intervals for selected GSs and STs that are significantly altered in the brainstem of rats during aging. Data are shown as Mean ± SEM (standard error of mean).

In this study, we observed changes in the levels of various ST species and GM1a gangliosides during aging (Table S2). Results from significance testing are plotted in Figure 3A as a heatmap and in Figure 3B as volcano plots comparing old and young healthy animals aged 12 months and 14 months.

In general, we observed significant alterations in the levels of several ST and dihexosyl-ST species in aging. Most of the altered STs followed a gradually decreasing trend during aging (ST t36:1, ST d42:1, ST d36:1, ST d34:1, dihexST d36:1, dihexST d42:3, dihexST d38:1, and others). In contrast, two ST species, namely ST d43:1 and ST t42:2 followed a rising trend. Overall, gangliosides were not significantly influenced by progressing age except for GM1a d42:2 and GM1a d40:2. There was a noticeable rising trend, and both were significantly upregulated in old rats compared to young ones (Figure 3C).

### 2.3. Glycosphingolipid Screening in an SHR24 Rat Model for Tauopathy

**Figure 4:**
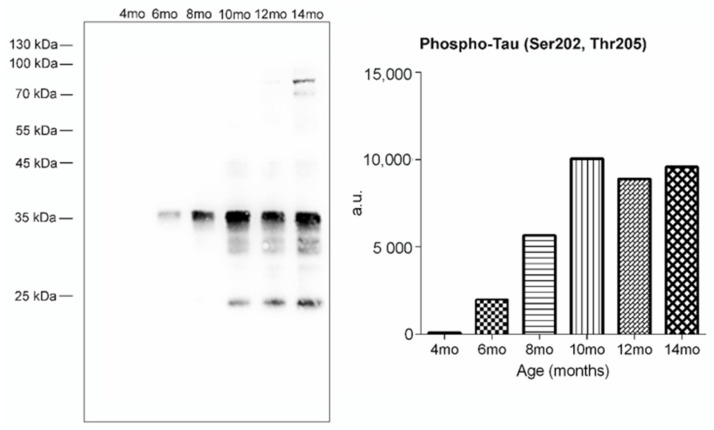
Ontogenesis analysis of paired helical filament load in the SHR24 brainstem. Progressive increase in the levels of sarkosyl-insoluble tau protein complexes in the brainstem of SHR24 rats. Ontogenesis of sarkosyl-insoluble tau complexes in aging rats was monitored by Western blot analysis using phosphorylation-dependent anti-tau AT8 antibodies.

Next, we aimed to investigate the impact of tau-mediated neurodegeneration on the GSL profile in the transgenic rat model. We evaluated the presence of neurofibrillary pathology in the brainstem of SHR24 transgenic rats and its ontogenesis throughout the aging process. Western blot analysis using phosphorylation-dependent anti-tau AT8 antibody revealed a gradual increase of sarkosyl-insoluble tau protein complexes in the brainstem of SHR24 transgenic rats. The neurofibrillary pathology in brain tissue is already present in 6-month-old animals. The levels of sarkosyl-insoluble tau culminated in 10-month-old animals and remained more or less on the same level in 12 and 14-month-old animals (Figure 4).

**Figure 5:**
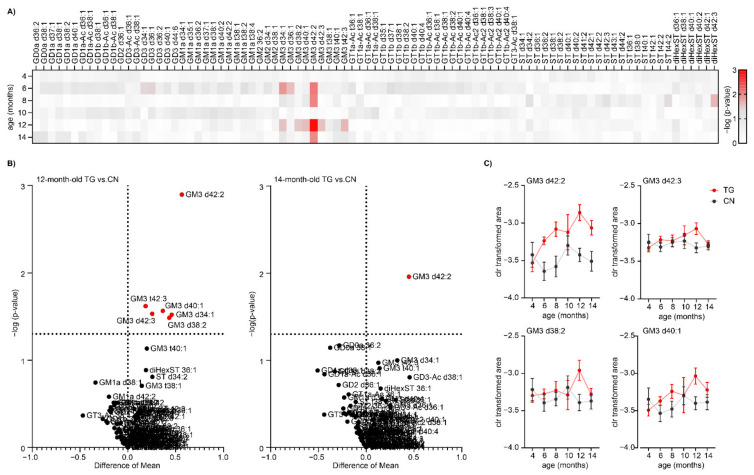
Increased levels of GM3 ganglioside species in the brainstem of SHR24 transgenic rats. (**A**) Heatmap showing results from significance testing as -log10(*p*-value) (**B**) Volcano plots show the results from the statistical analysis of the GSL profile in 12-month-old and 14-month-old TG vs. CN animals. The dashed line represents a p-value threshold of 0.05. (**C**) Confidence intervals of selected gangliosides from the GM3 class that were significantly altered in the brainstem of SHR24 rats when compared to controls. Data are shown as Mean ± SEM.

**Figure 6:**
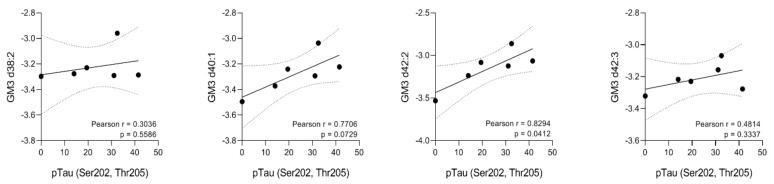
Correlation analysis between the most significantly upregulated GM3 ganglioside species and the amount of aggregated forms of tau protein in brain tissue. Pearson’s r was used for the calculation of correlation coefficient.

The results from PCA and PLS-DA suggested no significant changes between SHR24 transgenic rats and age-matched controls (Figure S1). However, univariate statistical analysis identified changes in the levels of several ganglioside species from the GM3 class as shown on a heatmap in Figure 5A. The volcano plot in Figure 5B shows the results from significance testing for 12-month-old animals which yielded the most significant changes in GSL levels. We found changes in the levels of GM3 d38:2, d40:1, d42:2, and GM3 d42:3 between 12-month-old TG and CN animals. Statistically significant lipids are separated by a dashed line. Figure 5B also shows the volcano plot of 14-month-old TG vs. CN mice, where GM3 d42:2 ganglioside was found elevated in TG mice relative to CN mice. The levels of these GM3 species followed a rising trend in TG mice, however, the change was most pronounced in 12-month-old animals. In 14-month-old animals, the detected levels in TG animals dropped to the levels of control animals. In transgenic animals, we have found elevated levels of GM3 d42:2 ganglioside with a increasing trend from 8 months of age (Figure 5C). Correlation analysis of GM3 class gangliosides showed that GM3 d42:2 significantly correlated with the amount of aggregated forms of tau protein in brain tissue (Figure 6).

We next assessed the potential role of astrocyte activation on the GM3 levels. We detected a significant increase in glial fibrillary acidic protein (GFAP) in the brain stem of SHR24 animals in comparison to the controls (SHR: 15.05 ± 1.39; SHR24: 24.53 ± 2.68; *n* = 7). The activation of astrocytes correlates with tau pathology (Figure S3). We were interested in studying if neurofibrillary degeneration aggravates the infiltration of T cells into the CNS. Therefore, we quantified the number of CD4+ and CD3+ cells. We observed an increase in the amount of CD4+ and CD3+ cells in the brainstem of tau transgenic animals (Figure S4).

## 3. Discussion

As essential components of membrane microdomains, GSLs play a crucial role in key cellular functions. However, GSL analysis in biological material is challenging and the exact brain GSL composition is still under investigation. Due to the large diversity in glycan and fatty acyl composition, many GSL species often remain undetected by current analytical approaches. In this study, we performed a selective screening of acidic GSLs in rat brain tissue, focusing on gangliosides (GS) and sulfatides (ST). We next examined GSL species in aging SHR animals and the SHR24 tau transgenic rat model. We first screened rat brain tissue for possible unknown lipid species and revealed two previously unknown GS subclasses with distinct glycans: GT1b-Ac2 and GQ1b-Ac2. Glycan moieties of these lipid subclasses were identified based on their specific fragmentation patterns. All GSLs that could be detected and reliably quantified in brain tissue were analyzed using LC-HRMS analysis.

During aging, significant changes in brain lipid composition occur. In our study, we observed significant alterations in the levels of several sulfatide species during aging. These were gradually decreasing with progressing age, which is in line with previous findings [16]. Sulfatides are predominantly synthesized in oligodendrocytes, enriched in myelin [17,18], and contribute to the maintenance and stability of myelin structure. Sulfatide depletion causes a gradual deterioration of myelin with age [19]. It has been proposed that this may be associated with dysregulated lipoprotein metabolism and aberrant lipid trafficking [20]. We observed no alterations in ST levels in tau transgenic animals. Numerous studies report the depletion of sulfatides in AD animal models [7] and AD brain tissue [9,20,21,22]. Our results and recent evidence [23] suggest that tau pathology is not directly involved in sulfatide depletion in AD.

The ganglioside profile was not significantly affected during the aging process, except for the GM1a ganglioside subclass. Elevated levels of GM1a d42:2 ganglioside were detected in our experiment. This particular subclass is well known for its neuroprotective and neuritogenic properties [24,25]. Recent evidence suggests that neuronal cells accumulate GM1 as a result of neuronal injury [26] while exogenous administration of GM1 protects against neurotoxicity and prevents neuronal damage [25,27]. It is important to note that both GM1a (d18:1-24:1) and (d20:1-22:1) could contribute to GM1a d42:2 signal. No previous study reported dysregulated levels of either of these specific lipids in this context, however an age-dependent increase in the d20:1/d18:1 ratio in gangliosides was previously reported [28,29]. Thus, the rise in GM1a d42:2 is probably due to the elevated ratio of d20:1/18:1, but further experiments would be necessary to confirm these results.

The levels of GM1a gradually rise with progressing age which may be a result of activated intrinsic neuroprotective responses. On the other hand, in the aged transgenic animals, no changes in the levels of GM1 were found. This may be associated with dysfunction in ganglioside-related neuroprotective processes.

The implication of gangliosides in AD has been previously reported [30,31,32]. Ganglioside species have been found to bind to different Aβ mutations and accelerate the assembly of amyloid fibrils [33,34]. In addition, a possible role of gangliosides in tau pathology has been implicated in progressive supranuclear palsy and Pick´s disease [35,36]. In our study, we found that the presence of tau pathology in neuronal tissue results in upregulated levels of the GM3 ganglioside subclass, with no specific pattern concerning the length and saturation of fatty acyls. In the present study, increased levels of GM3 correlate with tau pathology. Similar findings were observed in the entorhinal cortex of late-onset AD post-mortem brain tissue and transgenic familial AD mouse models [37,38]. This may be a result of a neuroinflammatory process or a higher rate of apoptosis. Using immunohistochemical staining we detected the presence of activated astrocytes and the transmigration of CD3+ and CD4+ T-cells in areas with neurofibrillary pathology. Thus, neuroinflammation induced by tau pathology is likely responsible for the increase of GM3 in our transgenic model.

According to these facts it can be suggested that acidic glycosphingolipids could play an important role in both aging and neurodegeneration and may be implicated in a wide range of neuronal processes. However, more detailed and well conducted studies are needed to bring clearer answers.

## 4. Materials and Methods

### 4.1. Animals

All LC-MS experiments were performed on male transgenic SHR24 rats expressing human truncated tau aa151-391/3R. As controls, we used age-matched non-transgenic animals (SHR). Animals included in LC-MS studies (131 in total) were 4, 6, 8, 10, 12, and 14 months old.

Animals used in this work were bred in the in-house animal facility of the Institute of Neuroimmunology of the Slovak Academy of Sciences in Bratislava. All animals were housed under standard laboratory conditions with free access to water and food and were kept under diurnal lighting conditions (12 h light/dark cycles with light starting at 7 a.m.). All animal experiments were performed according to the institutional animal care guidelines and in accordance with international standards (Animal Research: Reporting of In Vivo Experiments guidelines), and approved by the State Veterinary and Food Administration of the Slovak Republic and by the Ethics Committee of the Institute of Neuroimmunology, Slovak Academy of Sciences. Efforts were made to minimize the number of animals utilized and to limit discomfort, pain, or any other suffering of the experimental animals utilized in this study.

Animals were deeply anesthetized with tiletamine-zolazepam/xylazine anesthesia (4/2 mg/kg). Brain tissue was cut sagittally, medulla oblongata was separated, transferred into a separate tube, flash-frozen immediately in liquid nitrogen, and stored at −80 °C.

### 4.2. Chemicals and Reagents

Acetonitrile, methanol (HPLC/MS grade), chloroform (HPLC grade ≥99.8%, stabilized by amylene), ammonium acetate, and acetic acid were purchased from Sigma Aldrich (St. Louis, MO, USA), total ganglioside extract from the porcine brain was obtained from Avanti Polar Lipids (Alabaster, AL, USA). Deionized water was prepared from a Milli-Q water purification system (Millipore, Molsheim, France).

### 4.3. Sample Preparation

Tissue samples were prepared using a previously published protocol, slightly adjusted for the scale of the analysis [15]. Suitability of the modified extraction protocol was proven by a comparative extraction and reproducibility test (Supplementary Materials—Optimization of the sample preparation protocol, Figure S2 and Table S3).

Frozen tissue samples were cut on a frozen thermal block for a final weight of 15 mg ± 5 mg. Stainless steel beads and 1.5 mL of chloroform/methanol mixture (2:1 *v*/*v*) were added and the mixture was homogenized using a FastPrep-24 homogenizer (60 s, 5 m/s). After homogenization, 300 µL of deionized water was added and the mixture was vortexed and subsequently shaken for 15 min. After that, samples were centrifuged (4600 rpm, 10 min, room temperature) and the upper aqueous layer was collected and evaporated in a vacuum evaporator Savant SpeedVac SPD111V (Thermo Fisher Scientific, Waltham, MA, USA). Dried samples were reconstituted in 1 mL of deionized water, vortexed, and purified using a solid-phase extraction Sep-Pak tC18 100 mg 96-well plate (Waters, Milford, MA, USA). Methanolic eluates were dried again using a vacuum evaporator. Finally, dried samples were reconstituted in 100 µL of 90% acetonitrile and transferred into vials for LC-MS analysis. The QC samples were prepared by pooling 5 µL of each sample.

### 4.4. UHPLC-Q-TOF Analysis

A Waters (Waters, Milford, MA, USA) Synapt G2-Si QTOF mass spectrometer with electrospray (ESI) source coupled with an ACQUITY UPLC chromatographic system was used. Data were acquired using MassLynx v4.2 software (Waters, Milford, MA, USA). An Ascentis Si column 2.1 mm × 150 mm, 3 µm (Sigma Aldrich, St. Louis, MO, USA) was used for separation with a flow rate of 0.3 mL/min, an injection volume of 1 µL, and the column temperature was maintained at 40 °C. Mobile phase A consisted of 10 mM ammonium acetate (pH 6.1) adjusted by acetic acid and mobile phase B consisted of acetonitrile with the same amount of acetic acid needed for adjusting mobile phase A. Starting gradient conditions were 0 min: 87.7% B, then slowly rising to 77.9% B for 15 min and re-equilibration from 15.01 to 25 min. Mass spectrometric conditions were as follows: sensitivity mode, negative ion mode, mass range *m*/*z* 300–2000, capillary voltage 2.5 kV, sampling cone 20 V, source offset 90 V, source temperature 150 °C, desolvation temperature 250 °C, cone gas flow 50 L/h, desolvation gas flow 300 L/h, and nebulizer gas flow 4.0 bar. Leucine enkephaline was used as the lock mass calibrant. MS/MS experiments were performed on the transfer cell with the collision energy ramp.

### 4.5. Immunohistochemical Staining

Animals were deeply anesthetized with tiletamine-zolazepam/xylazine anesthesia (4/2 mg/kg) and perfused intracardially with PBS. The brain was removed and embedded in a cryostat embedding medium (Leica, Wetzlar, Germany) and frozen above the surface of liquid nitrogen. Brain sections 12 μm-thick were cut on a cryomicrotome (Leica CM 1850, Leica, Wetzlar, Germany), affixed onto poly-L-lysine coated slides, and left to dry at room temperature for 1 h. Sections were fixed for 15 min in 4% paraformaldehyde and blocked for 60 min in blocking solution (DAKO, Mississauga, ON, Canada). Sections were incubated with primary antibodies: polyclonal rabbit anti-rat GFAP (Thermo Fisher Scientific) and monoclonal mouse anti-rat pSer202/pT205 (Thermo Fisher Scientific), polyclonal rabbit anti-rat CD4 (1:200; Novus Biologicals, Centennial, CO, USA), polyclonal rabbit anti-rat CD3 (1:200; Novus Biologicals, Centennial, CO, USA). After washing, sections were incubated for 1 h in secondary antibodies: goat anti-rabbit or goat anti-mouse AlexaFluor488/546 (1:1000; Invitrogen Life Technologies, Carlsbad, CA, USA). Sections were mounted (Vector Laboratories, Burlingame, CA, USA) and examined using an LSM 710 confocal microscope (Zeiss, Jena, Germany).

### 4.6. Preparation of Sarkosyl-Insoluble PHF-tau from Transgenic Rat Brains

For isolation of sarkosyl-insoluble tau we used 4, 6, 8, 10, 12 and 14-month-old transgenic animals (*n* = 3). Briefly, 100 mg of the brainstem from transgenic rat brain enriched in PHF-tau were dissected, cleaned of blood vessels and meninges, and used as a starting material. The brain tissue was homogenized on ice in 10 volumes of ice-cold SL buffer (20 mM Tris- HCl, pH 7.4, 800 mM NaCl, 1 mM EGTA, 1mM EDTA, 0.5% β-mercaptoethanol, 10% sucrose, 1× protease inhibitors Complete, EDTA-free). The homogenate was centrifuged for 20 min at 20,000× *g*. Solid sarkosyl (Sigma-Aldrich, St. Louis, MO, USA) was added to the supernatant to achieve a 1% concentration. The samples were stirred for 1 h at room temperature (RT). The samples were spun for 1 h at RT in a Beckman ultracentrifuge using SW 120 Ti rotor (Beckman Coulter, Inc., Brea, CA, USA) at 100,000× *g*. After sarkosyl extraction and ultracentrifugation, pellets containing PHF-tau were dissolved in 1× SDS sample loading buffer and boiled for 5 min. Samples were determined by following Western blot analysis.

### 4.7. Biochemical Western Blot Analysis

Samples were separated onto 10% SDS-polyacrylamide gels and transferred to a nitrocellulose membrane in 10 mM N-cyclohexyl-3-aminopropanesulfonic acid (CAPS, pH 11, Roth, Karlsruhe, Germany). The membranes were blocked in 5% milk in Tris-buffered saline with 0.1% Tween 20 (Sigma-Aldrich) (TBS-T, 137 mM NaCl, 20 mM Tris base, pH 7.4, 0.1% Tween 20) for 1 h and incubated with primary antibody, monoclonal mouse anti-rat pSer202/pT205 overnight at 4 °C. Membranes were incubated with horseradish peroxidase (HRP)-conjugated secondary antibody in TBS-T (1:2000, Dako, Glostrup, Denmark) for 1 h at room temperature. Immunoreactive proteins were detected by chemiluminescence (SuperSignal West Pico Chemiluminescent Substrate, Thermo Scientific, Pittsburgh, PA, USA) and the signals were digitized by Image Reader LAS-3000 (FUJIFILM, Bratislava, Slovakia).

### 4.8. Data Analysis

Raw mass spectra were processed using Skyline software v20.1.0.155 (MacCoss lab, University of Washington, Seattle, WA, USA) [39]. Lipid species were detected according to accurate *m*/*z* values with a mass tolerance of ±10 ppm. After isotopic correction of signal responses, data were processed and statistically evaluated in R software (version 3.5.0) using a homemade Metabol package [40,41]. At first, the quality control-based locally estimated smoothing signal correction (LOESS) was applied on each dataset. The coefficient of variation (CV) based on QC samples was calculated, and compounds with a CV higher than 30% were excluded from further processing. In the case of the brain, correction of datasets to sample weight was performed. The data were transformed by natural logarithm (ln) and main centering was applied. Univariate and multivariate analyses were used for statistical evaluation and visualization of the data.

## Data Availability

The mass spectrometry data have been deposited to a public repository (https://doi.org/10.6084/m9.figshare.16819063.v1). The data presented in this study are available on request from the corresponding author.

## Supplementary Materials

The following are available online at https://www.mdpi.com/article/10.3390/ijms222212581/s1.
