# Supplementary material for: GM3 Ganglioside Linked to Neurofibrillary Pathology in a Transgenic Rat Model for Tauopathy
Source: Int J Mol Sci. 2021 Nov 22;22(22):12581. doi: 10.3390/ijms222212581 (PMC8622195; doi:10.3390/ijms222212581)
Supplement: Supplementary file 1 [file ijms-22-12581-s001.zip › ijms-1446965-sm.pdf]

International Journal of
Molecular Sciences

MDPI

Article

GM3 Ganglioside Linked to Neurofibrillary Pathology in a Transgenic Rat Model for Tauopathy

Dominika Olešová [1,2], Petra Majerová [1], Roman Hájek [3], Juraj Piešťanský [4], Radana Brumarová [5], Alena Michalicová [1], Bernadeta Jurkanin [1], David Friedecký [5] and Andrej Kováč [1,*]

[1] Institute of Neuroimmunology, Slovak Academy of Sciences, Dúbravská cesta 9, 84510 Bratislava, Slovak Republic; dominika.olesova@savba.sk (D.O.); petra.majerova@savba.sk (P.M.); alena.michalicova@savba.sk (A.M.); bernadeta.valachova@savba.sk (B.J.)

[2] Laboratory of Biomedical Microbiology and Immunology, University of Veterinary Medicine and Pharmacy, 04181 Kosice, Slovak Republic

[3] Waters Corporation, Stamford Avenue, Altrincham Road, Wilmslow SK9 4AX, United Kingdom; roman_hajek@waters.com (R.H.)

[4] Department of Pharmaceutical Analysis and Nuclear Pharmacy, Faculty of Pharmacy, Comenius University in Bratislava, Odbojarov 10, 83232 Bratislava, Slovak Republic; piestansky@fpharm.uniba.sk (J.P.)

[5] Laboratory for Inherited Metabolic Disorders, Department of Clinical Biochemistry, University Hospital Olomouc, and Faculty of Medicine and Dentistry, Palacký University Olomouc, I. P. Pavlova 6, 77900 Olomouc, Czech Republic; radana.karlikova@gmail.com (R.B.), david.friedecky@upol.cz (D.F.)

* Correspondence: andrej.kovac@savba.sk; Tel.: +421-2-54788100

(Supplementary Material)

Table of contents

Table S1. List of glycosphingolipids identified in pooled rat brain tissue using HILIC/ESI-MS/MS method.

Table S2. Statistical analysis of data from GSL screening in aging rats and transgenic rat model for tau pathology.

Table S3. Results from reproducibility testing.

Figure S1. Principal component analysis (A) and Orthogonal-least square – discriminant analysis (B) of GSL screening data.

Figure S2. Comparison of relative peak areas for different extraction volumes, shown for major individual gangliosides GD1a d36:1, GD1b d36:1, GM1a d36:1, GM3 d36:1, and ST d36:1.

Figure S3. Immunostaining pattern of astrocytes and tau pathology in SHR-24 tau transgenic and cotrol animals (SHR).

Figure S4. CD3+ T-cell and CD4+ T-cell occurrence in SHR-24 transgenic rat model for tauopathies compare to control animal (SHR).

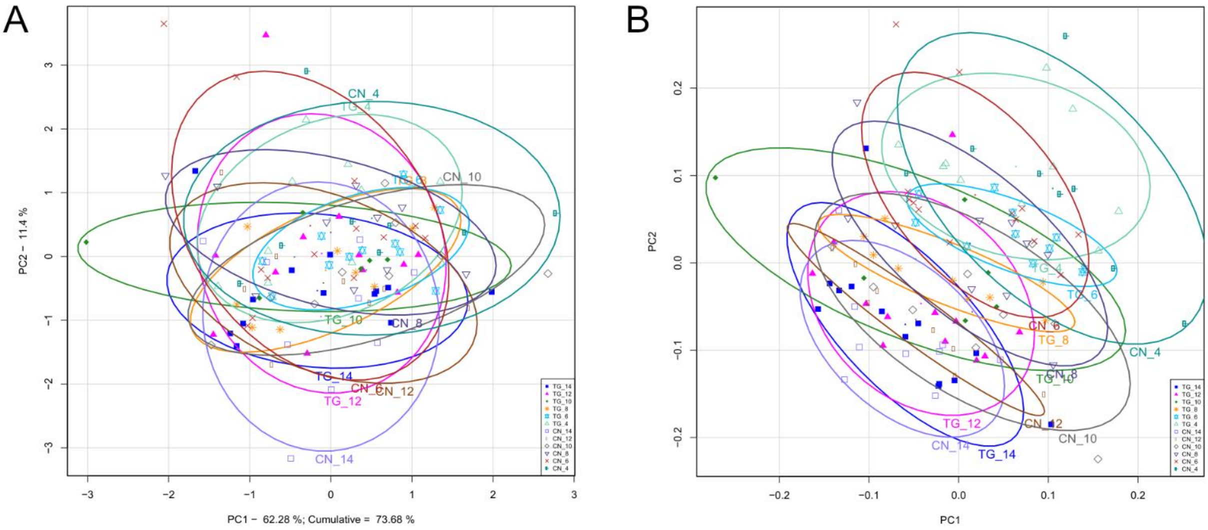

Figure S1. Principal component analysis (A) and Orthogonal-least square – discriminant analysis (B) of GSL screening data.

Optimization of the sample preparation protocol

The original extraction procedure [1] was modified in order to improve the sample throughput and to minimize the consumption of the organic solvents. Different amounts of extraction solvent were tested and compared with the original method to verify if the yields of the adjusted method are satisfactory. Results from extraction method comparison are shown in Figure S2. The obtained results showed acceptable extraction efficiency of the modified protocol. Moreover, the reproducibility of the modified extraction protocol was investigated. It was tested on 4 separately prepared samples, each with 3 injections. Results from reproducibility testing were satisfactory (RSD ≤ 18%) and are shown in Table S3.

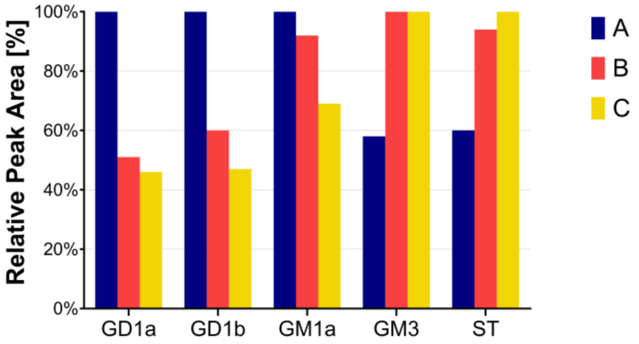

Figure S2. Comparison of relative peak areas for different extraction volumes, shown for major individual gangliosides GD1a d36:1, GD1b d36:1, GM1a d36:1, GM3 d36:1, and ST d36:1. Method A - 6 ml of extraction solution, Method B - 3 ml of extraction solution, and Method C - 1.5 ml of extraction solution.

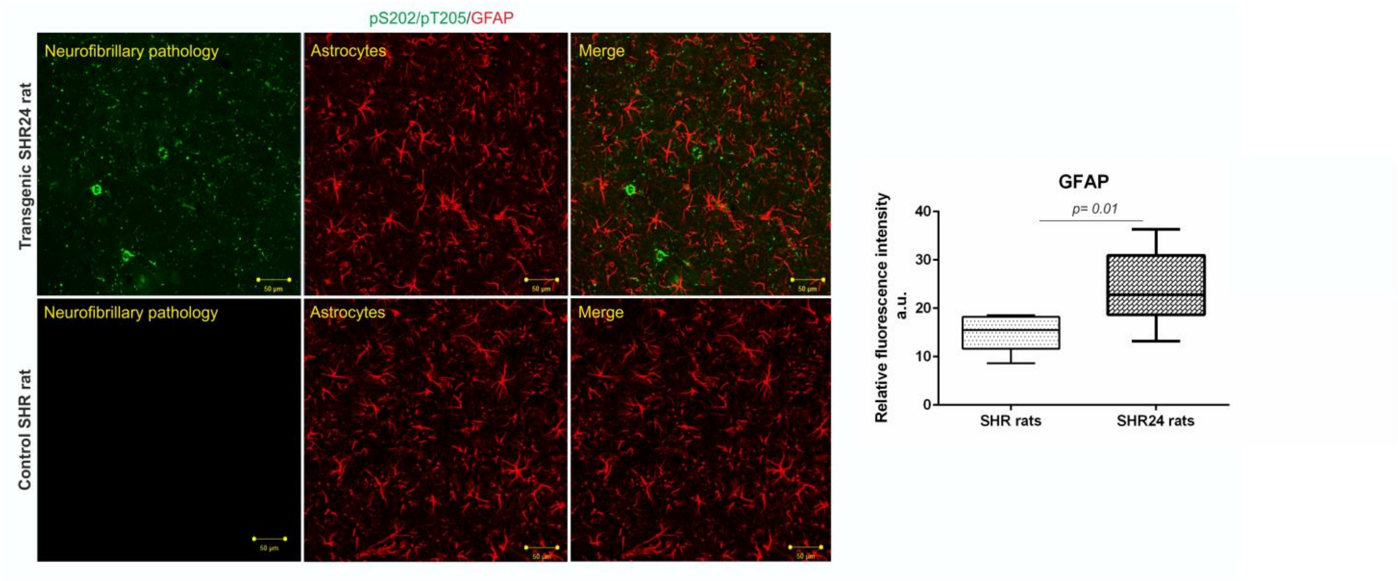

Figure S3. Immunostaining pattern of astrocytes and tau pathology in SHR-24 tau transgenic and control animals (SHR). Confocal microscopy showed that astrocytes stained with GFAP (red color) were distributed throughout the brain areas affected by neurofibrillary lesions immunolabelled with pSer202/pThr205 (AT8, green color).

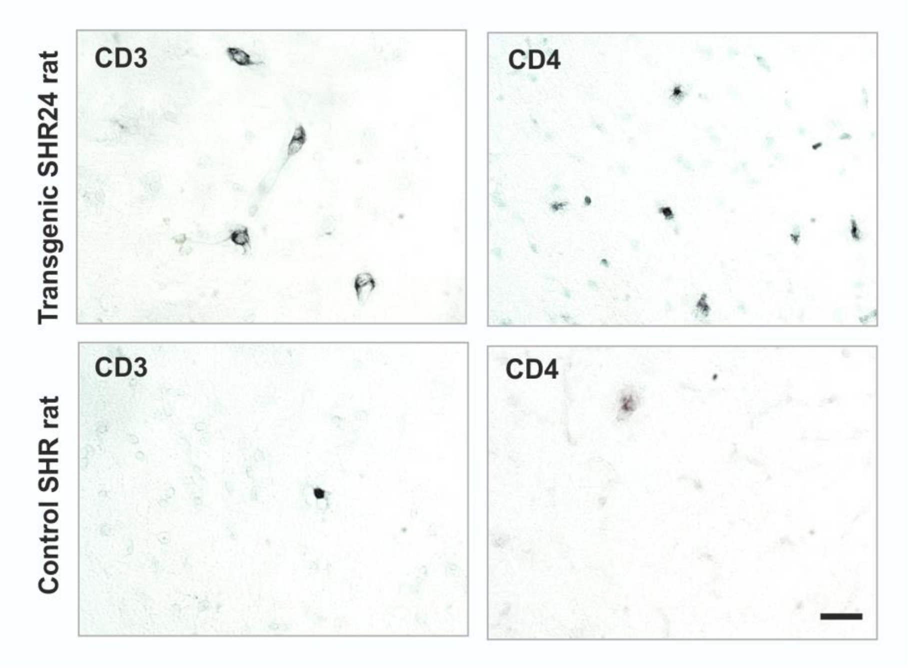

Figure S4. CD3+ T-cell and CD4+ T-cell occurrence in SHR-24 transgenic rat model for tauopathies compare to control animals (SHR). Scale bar 20 μm, n = 3.

Table S1. List of glycosphingolipids identified in pooled rat brain tissue using HILIC/ESI-MS/MS method.

Class	Monoisotopic mass	Lipid species level	Elemental composition	Adduct	Theoretical m/z	Experimental m/z	Mass error [ppm]	RT [min.]	RIC peak height
**GD0a**	2038.0212	GD0a d36:2	C92H159N5O44	[M-2H]	1018.0106	1018.0069	-3.63	7.50	4.84E+04
**GD0a**	2040.0370	GD0a d36:1	C92H161N5O44	[M-2H]	1019.0185	1019.0154	-3.04	7.45	8.66E+03
**GD0a**	2066.0526	GD0a d38:2	C94H163N5O44	[M-2H]	1032.0263	1032.0160	-9.98	7.38	3.90E+03
**GD0a**	2068.0827	GD0a d38:1	C94H165N5O44	[M-2H]	1033.0341	1033.0284	-5.52	7.40	1.84E+04
**GD1a**	1834.9564	GD1a d36:2	C84H146N4O39	[M-2H]	916.4709	916.4633	-8.29	6.46	6.60E+04
**GD1a**	1836.9721	GD1a d36:1	C84H148N4O39	[M-2H]	917.4788	917.4767	-2.29	6.43	1.02E+06
**GD1a**	1850.9877	GD1a d37:1	C85H150N4O39	[M-2H]	924.4866	924.4915	5.30	6.36	2.05E+04
**GD1a**	1862.9877	GD1a d38:2	C86H150N4O39	[M-2H]	930.4866	930.4889	2.47	6.33	3.77E+04
**GD1a**	1865.0034	GD1a d38:1	C86H152N4O39	[M-2H]	931.4944	931.4911	-3.54	6.33	3.94E+05
**GD1a**	1893.0347	GD1a d40:1	C88H156N4O39	[M-2H]	945.5101	945.5026	-7.93	6.26	2.20E+04
**GD1a-Ac**	1876.9670	GD1a-Ac d36:2	C86H148N4O40	[M-2H]	937.4762	937.4800	4.05	4.8	1.37E+04
**GD1a-Ac**	1878.9826	GD1a-Ac d36:1	C86H150N4O40	[M-2H]	938.4840	938.4766	-7.89	4.9	1.59E+05
**GD1a-Ac**	1907.0139	GD1a-Ac d38:1	C88H154N4O40	[M-2H]	952.4997	952.4944	-5.56	4.8	1.60E+05
**GD1b**	1834.9564	GD1b d36:2	C84H146N4O39	[M-2H]	916.4709	916.4745	3.93	9.08	2.58E+04
**GD1b**	1836.9721	GD1b d36:1	C84H148N4O39	[M-2H]	917.4788	917.4771	-1.85	9.05	3.95E+05
**GD1b**	1862.9877	GD1b d38:2	C86H150N4O39	[M-2H]	930.4866	930.4919	5.70	8.98	2.25E+04
**GD1b**	1865.0034	GD1b d38:1	C86H152N4O39	[M-2H]	931.4944	931.4916	-3.01	8.93	2.21E+05
**GD1b**	1893.0347	GD1b d40:1	C88H156N4O39	[M-2H]	945.5101	945.5074	-2.86	8.86	1.50E+04
**GD1b-Ac**	1876.9670	GD1b-Ac d36:2	C86H148N4O40	[M-2H]	937.4762	937.4720	-4.48	6.79	2.14E+04
**GD1b-Ac**	1878.9826	GD1b-Ac d36:1	C86H150N4O40	[M-2H]	938.4840	938.4808	-3.41	6.80	3.20E+05
**GD1b-Ac**	1907.0139	GD1b-Ac d38:1	C88H154N4O40	[M-2H]	952.4997	952.4960	-3.88	6.68	2.16E+05
**GD2**	1674.9192	GD2 d36:1	C78H138N4O34	[M-2H]	836.4524	836.4514	-1.20	7.77	3.62E+04
**GD2**	1702.9505	GD2 d38:1	C80H142N4O34	[M-2H]	850.4680	850.4666	-1.65	7.70	7.79E+03
**GD3**	1443.8086	GD3 d34:1	C68H121N3O29	[M-2H]	720.8970	720.8930	-5.55	5.32	3.32E+03
**GD3**	1469.8242	GD3 d36:2	C70H123N3O29	[M-2H]	733.9048	733.8992	-7.63	5.26	5.23E+03
**GD3**	1471.8399	GD3 d36:1	C70H125N3O29	[M-2H]	734.9127	734.9106	-2.86	5.26	1.13E+05
**GD3**	1497.8555	GD3 d38:2	C72H127N3O29	[M-2H]	747.9205	747.9182	-3.08	5.17	6.29E+03
**GD3**	1499.8712	GD3 d38:1	C72H129N3O29	[M-2H]	748.9283	748.9256	-3.61	5.22	5.45E+04
**GD3**	1527.9025	GD3 d40:1	C74H133N3O29	[M-2H]	762.9440	762.9379	-8.00	5.14	6.20E+03
**GD3**	1543.8399	GD3 d42:7	C76H125N3O29	[M-2H]	770.9121	770.9117	-0.52	5.14	1.38E+04
**GD3**	1573.8868	GD3 d44:6	C78H131N3O29	[M-2H]	785.9356	785.9395	4.96	5.10	9.28E+03
**GD3-Ac**	1513.8504	GD3-Ac d36 1	C72H127N3O30	[M-2H]	755.9174	755.9128	-6.09	3.51	9.46E+04
**GD3-Ac**	1541.8817	GD3-Ac d38 1	C74H131N3O30	[M-2H]	769.9330	769.9307	-2.99	3.40	1.03E+05
**GM1a**	1517.8454	GM1a d34:1	C71H127N3O31	[M-H]	1516.8381	1516.8370	-0.73	5.27	1.88E+05
**GM1a**	1531.8610	GM1a d35:1	C72H129N3O31	[M-H]	1530.8537	1530.8536	-0.07	5.21	3.24E+04
**GM1a**	1542.8532	GM1a d36:2	C73H128N3O31	[M-H]	1542.8537	1542.8540	0.19	5.19	6.89E+05
**GM1a**	1545.8767	GM1a d36:1	C73H131N3O31	[M-H]	1544.8694	1544.8699	0.32	5.19	3.88E+06

**GM1a**	1559.8923	GM1a d37:1	C74H133N3O31	[M-H]	1558.8845	1558.8768	-4.94	5.12	5.54E+04
**GM1a**	1561.8716	GM1a t36:1	C73H131N3O32	[M-H]	1560.8637	1560.8784	9.42	5.10	1.99E+04
**GM1a**	1563.8872	GM1a t36:0	C73H133N3O32	[M-H]	1562.8794	1562.8784	-0.64	5.21	1.38E+04
**GM1a**	1571.8923	GM1a d38:2	C75H133N3O31	[M-H]	1570.8850	1570.8843	-0.45	5.07	2.05E+05
**GM1a**	1573.9080	GM1a d38:1	C75H135N3O31	[M-H]	1572.9007	1572.8992	-0.95	5.09	4.16E+05
**GM1a**	1583.8559	GM1a t38:4	C75H129N3O32	[M-H]	1582.8481	1582.8350	-8.28	5.17	1.18E+04
**GM1a**	1587.8872	GM1a t38:2	C75H133N3O32	[M-H]	1586.8794	1586.8776	-1.13	5.21	1.39E+04
**GM1a**	1589.9029	GM1a t38:1	C75H135N3O32	[M-H]	1588.8950	1588.8857	-5.85	5.32	5.59E+04
**GM1a**	1599.9236	GM1a d40:2	C77H137N3O31	[M-H]	1598.9163	1598.9095	-4.25	4.99	1.44E+04
**GM1a**	1627.9549	GM1a d42:2	C79H141N3O31	[M-H]	1626.9476	1626.9331	-8.91	4.91	5.47E+04
**GM2**	1355.7925	GM2 d34:1	C65H117N3O26	[M-H]	1354.7853	1354.7958	7.75	3.91	1.56E+04
**GM2**	1381.8082	GM2 d36:2	C67H119N3O26	[M-H]	1380.8009	1380.7960	-3.55	3.80	2.62E+04
**GM2**	1383.8238	GM2 d36:1	C67H121N3O26	[M-H]	1382.8166	1382.8152	-1.01	3.83	1.48E+05
**GM2**	1409.8395	GM2 d38:2	C69H122N3O26	[M-H]	1408.8322	1408.8356	2.41	3.72	8.10E+03
**GM2**	1411.8551	GM2 d38:1	C69H125N3O26	[M-H]	1410.8479	1410.8452	-1.91	3.72	2.95E+04
**GM2**	1465.9021	GM2 d42:2	C73H131N3O26	[M-H]	1464.8948	1464.8883	-4.44	3.64	9.63E+03
**GM3**	1152.7132	GM3 d34:1	C57H104N2O21	[M-H]	1151.7059	1151.7040	-1.65	2.28	2.27E+05
**GM3**	1166.6924	GM3 t34:2	C57H102N2O22	[M-H]	1165.6851	1165.6936	7.29	2.26	1.96E+04
**GM3**	1168.7081	GM3 t34:1	C57H104N2O22	[M-H]	1167.7008	1167.6949	-5.05	2.21	9.63E+03
**GM3**	1178.7288	GM3 d36:2	C59H106N2O21	[M-H]	1177.7215	1177.7133	-6.96	2.23	5.53E+04
**GM3**	1180.7445	GM3 d36:1	C59H108N2O21	[M-H]	1179.7372	1179.7378	0.51	2.23	2.12E+06
**GM3**	1194.7237	GM3 t36:2	C59H106N2O22	[M-H]	1193.7164	1193.7220	4.69	2.20	2.43E+04
**GM3**	1198.6975	GM3 d38:6	C61H102N2O21	[M-H]	1197.6902	1197.6790	-9.35	2.09	4.10E+04
**GM3**	1206.7601	GM3 d38:2	C61H110N2O21	[M-H]	1205.7528	1205.7467	-5.06	2.18	4.24E+04
**GM3**	1208.7758	GM3 d38:1	C61H112N2O21	[M-H]	1207.7685	1207.7666	-1.57	2.20	1.42E+05
**GM3**	1222.7550	GM3 d39:1	C61H110N2O22	[M-H]	1221.7477	1221.7449	-2.29	2.09	2.80E+04
**GM3**	1224.7707	GM3 t38:1	C61H112N2O22	[M-H]	1223.7634	1223.7599	-2.86	2.09	1.25E+04
**GM3**	1234.7914	GM3 d40:2	C63H114N2O21	[M-H]	1233.7841	1233.7804	-3.00	2.14	2.95E+04
**GM3**	1236.8071	GM3 d40:1	C63H116N2O21	[M-H]	1235.7998	1235.8000	0.16	2.14	3.42E+04
**GM3**	1252.8020	GM3 t40:1	C63H116N2O22	[M-H]	1251.7947	1251.7939	-0.64	2.14	1.01E+04
**GM3**	1260.8071	GM3 d42:3	C65H116N2O21	[M-H]	1259.7998	1259.7882	-9.21	2.13	1.23E+04
**GM3**	1262.8227	GM3 d42:2	C65H118N2O21	[M-H]	1261.8154	1261.8209	4.36	2.11	1.05E+05
**GM3**	1276.8020	GM3 t42:3	C65H116N2O22	[M-H]	1275.7947	1275.8051	8.15	2.14	6.81E+03
**GQ1b-Ac**	2461.1735	GQ1b-Ac d36:1	C108H184N6O56	[M-3H]	819.3839	819.3812	-3.30	9.54	3.16E+04
**GQ1b-Ac**	2489.2048	GQ1b-Ac d38:1	C110H188N6O56	[M-3H]	828.7276	828.7278	0.24	9.47	2.72E+04
**GQ1b-Ac**	2461.1735	GQ1b-Ac t36:1	C108H184N6O56	[M-2H]	1229.5879	1229.5822	-4.64	9.49	2.13E+04
**GQ1b-Ac**	2489.2048	GQ1b-Ac t38:1	C110H188N6O56	[M-2H]	1243.5946	1243.5934	-0.96	9.34	1.45E+04
**GQ1b-Ac2**	2503.1840	GQ1b-Ac2 d36:1	C110H186N6O57	[M-3H]	833.3869	833.3834	-4.20	6.09	7.16E+04
**GQ1b-Ac2**	2531.2153	GQ1b-Ac2 d38:1	C112H190N6O57	[M-3H]	842.7306	842.7281	-2.97	6.01	1.01E+05
**GQ1b-Ac2**	2503.1840	GQ1b-Ac2 t36:1	C110H186N6O57	[M-2H]	1250.5842	1250.5796	-3.68	6.39	8.97E+04
**GQ1b-Ac2**	2531.2153	GQ1b-Ac2 t38:1	C112H190N6O57	[M-2H]	1264.5998	1264.5961	-2.93	6.23	5.99E+04
**GT1a**	2126.0518	GT1a d36:2	C95H163N5O47	[M-2H]	1062.0187	1062.0197	0.94	low int	low int
**GT1a**	2128.0675	GT1a d36:1	C95H165N5O47	[M-2H]	1063.0265	1063.0245	-1.88	low int	low int

**GT1a**	2156.0988	GT1a d38:1	C97H169N5O47	[M-2H]	1077.0421	1077.0439	1.67	9.30	9.94E+03
**GT1a-Ac**	2170.0781	GT1a-Ac d36:1	C97H167N5O48	[M-3H]	722.3521	722.3515	-0.83	7.30	1.83E+04
**GT1a-Ac**	2198.1094	GT1a-Ac d38:1	C99H171N5O48	[M-3H]	731.6958	731.6937	-2.87	7.26	1.67E+04
**GT1a-Ac**	2170.0781	GT1a-Ac d36:1	C97H167N5O48	[M-2H]	1084.0318	1084.0287	-2.86	7.30	4.51E+04
**GT1a-Ac**	2192.0624	GT1a-Ac d36:4	C99H165N5O48	[M-2H]	1095.0234	1095.0209	-2.28	7.33	3.47E+03
**GT1a-Ac**	2198.1094	GT1a-Ac d38:1	C99H171N5O48	[M-2H]	1098.0474	1098.0432	-3.82	7.25	4.75E+04
**GT1a-Ac**	2220.0937	GT1a-Ac d40:4	C101H169N5O48	[M-2H]	1109.0395	1109.0294	-9.11	7.20	5.77E+03
**GT1a-Ac**	2226.1407	GT1a-Ac d40:1	C101H175N5O48	[M-2H]	1112.0631	1112.0596	-3.15	low int	low int
**GT1b**	2100.0362	GT1b d34:1	C93H161N5O47	[M-2H]	1049.0108	1049.0208	9.53	11.07	4.46E+03
**GT1b**	2114.0518	GT1b d35:1	C94H163N5O47	[M-2H]	1056.0186	1056.0211	2.37	11.02	4.60E+03
**GT1b**	2126.0518	GT1b d36:2	C95H163N5O47	[M-2H]	1062.0187	1062.0201	1.32	10.92	1.23E+04
**GT1b**	2128.0675	GT1b d36:1	C95H165N5O47	[M-2H]	1063.0265	1063.0227	-3.57	10.94	1.76E+05
**GT1b**	2142.0831	GT1b d37:1	C96H167N5O47	[M-2H]	1070.0343	1070.0370	2.52	10.85	4.66E+03
**GT1b**	2150.0518	GT1b d38:4	C97H163N5O47	[M-2H]	1074.0187	1074.0085	-9.50	10.90	1.37E+04
**GT1b**	2154.0831	GT1b d38:2	C97H167N5O47	[M-2H]	1076.0343	1076.0287	-5.20	10.83	1.10E+04
**GT1b**	2156.0988	GT1b d38:1	C97H169N5O47	[M-2H]	1077.0421	1077.0382	-3.62	10.82	9.11E+04
**GT1b**	2166.0468	GT1b t38:4	C97H163N5O48	[M-2H]	1082.0156	1082.0072	-7.76	10.94	1.70E+04
**GT1b**	2178.0831	GT1b d40:4	C99H167N5O47	[M-2H]	1088.0340	1088.0370	2.76	10.80	6.79E+03
**GT1b**	2184.1301	GT1b d40:1	C99H173N5O47	[M-2H]	1091.0578	1091.0514	-5.87	10.80	8.87E+03
**GT1b-Ac**	2170.0781	GT1b-Ac d36:1	C97H167N5O48	[M-3H]	722.3521	722.3497	-3.32	9.25	6.16E+04
**GT1b-Ac**	2198.1094	GT1b-Ac d38:1	C99H171N5O48	[M-3H]	731.6958	731.6949	-1.23	9.20	7.13E+04
**GT1b-Ac**	2170.0781	GT1b-Ac td36:1	C97H167N5O48	[M-2H]	1084.0318	1084.0302	-1.48	9.29	2.93E+05
**GT1b-Ac**	2192.0624	GT1b-Ac d36:4	C99H165N5O48	[M-2H]	1095.0234	1095.0212	-2.01	9.29	2.04E+04
**GT1b-Ac**	2196.0937	GT1b-Ac d38:2	C99H169N5O48	[M-2H]	1097.0390	1097.0333	-5.20	9.25	1.51E+04
**GT1b-Ac**	2198.1094	GT1b-Ac d38:1	C99H171N5O48	[M-2H]	1098.0474	1098.0455	-1.73	9.20	3.31E+05
**GT1b-Ac**	2220.0937	GT1b-Ac d40:4	C101H169N5O48	[M-2H]	1109.0395	1109.0370	-2.25	9.18	2.43E+04
**GT1b-Ac**	2226.1407	GT1b-Ac d40:1	C101H175N5O48	[M-2H]	1112.0631	1112.0577	-4.86	9.10	2.10E+04
**GT1b-Ac2**	2212.0886	GT1b-Ac2 d36:1	C99H169N5O49	[M-2H]	1105.0365	1105.0367	0.18	7.89	2.91E+05
**GT1b-Ac2**	2226.0679	GT1b-Ac2 t36:2	C99H167N5O50	[M-2H]	1112.0261	1112.0345	7.55	7.89	6.03E+03
**GT1b-Ac2**	2234.0730	GT1b-Ac2 d38:4	C101H167N5O49	[M-2H]	1116.0287	1116.0311	2.15	7.90	1.28E+04
**GT1b-Ac2**	2236.0886	GT1b-Ac2 d38:3	C101H169N5O49	[M-2H]	1117.0365	1117.0269	-8.59	7.90	1.13E+04
**GT1b-Ac2**	2240.1199	GT1b-Ac2 d38:1	C101H173N5O49	[M-2H]	1119.0521	1119.0498	-2.06	7.82	5.01E+05
**GT1b-Ac2**	2262.1043	GT1b-Ac2 d40:4	C103H171N5O49	[M-2H]	1130.0443	1130.0419	-2.12	7.82	5.36E+04
**GT1b-Ac2**	2268.1512	GT1b-Ac2 d40:1	C103H177N5O49	[M-2H]	1133.0678	1133.0703	2.21	7.72	3.26E+04
**GT3-Ac**	1808.9408	GT3-Ac t35:0	C82H144N4O39	[M-2H]	903.4631	903.4644	1.44	6.50	1.71E+04
**GT3-Ac**	1822.9564	GT3-Ac t36:0	C83H146N4O39	[M-2H]	910.4709	910.4628	-8.90	6.50	9.90E+04
**GT3-Ac**	1832.9772	GT3-Ac d38:1	C85H148N4O38	[M-2H]	915.4813	915.4797	-1.75	6.50	8.07E+03
**ST**	777.5061	ST d34:2	C40H75NO11S	[M-H]	776.4988	776.4969	-2.45	0.88	1.52E+04
**ST**	779.5217	ST d34:1	C40H77NO11S	[M-H]	778.5144	778.5129	-1.93	0.88	1.34E+06
**ST**	805.5374	ST d36:2	C42H79NO11S	[M-H]	804.5301	804.5307	0.75	0.88	5.23E+05
**ST**	807.5530	ST d36:1	C42H81NO11S	[M-H]	806.5458	806.5486	3.47	0.88	8.82E+06
**ST**	821.5323	ST t36:2	C42H79NO12S	[M-H]	820.5250	820.5323	8.90	0.90	1.07E+05
**ST**	823.5479	ST t36:1	C42H81NO12S	[M-H]	822.5407	822.5460	6.44	0.87	1.08E+06

**ST**	833.5687	ST d38:2	C44H83NO11S	[M-H]	832.5614	832.5583	-3.72	0.88	2.35E+05
**ST**	835.5843	ST d38:1	C44H85NO11S	[M-H]	834.5770	834.5775	0.60	0.88	1.44E+06
**ST**	849.5636	ST t38:2	C44H83NO12S	[M-H]	848.5563	848.5593	3.54	0.87	9.82E+04
**ST**	851.5792	ST t38:1	C44H85NO12S	[M-H]	850.5720	850.5751	3.64	0.88	3.17E+05
**ST**	853.5949	ST t38:0	C44H87NO12S	[M-H]	852.5876	852.5822	-6.33	0.88	9.42E+04
**ST**	859.5843	ST d40:3	C46H85NO11S	[M-H]	858.5770	858.5768	-0.23	0.87	6.85E+04
**ST**	861.6000	ST d40:2	C46H87NO11S	[M-H]	860.5927	860.5952	2.90	0.87	1.80E+06
**ST**	863.6156	ST d40:1	C46H89NO11S	[M-H]	862.6083	862.6083	0.00	0.87	2.02E+06
ST	875.6156	ST d41:2	C47H89NO11S	[M-H]	874.6083	874.6072	-1.26	0.87	4.30E+05
**ST**	879.6105	ST t40:1	C46H89NO12S	[M-H]	878.6033	878.6058	2.85	0.88	2.05E+06
**ST**	881.6262	ST t40:0	C46H91NO12S	[M-H]	880.6189	880.6111	-8.86	0.88	4.25E+05
**ST**	887.6156	ST d42:3	C48H89NO11S	[M-H]	886.6083	886.6100	1.92	0.87	9.72E+05
**ST**	889.6313	ST d42:2	C48H91NO11S	[M-H]	888.6240	888.6270	3.38	0.87	2.24E+07
**ST**	891.6469	ST d42:1	C48H93NO11S	[M-H]	890.6396	890.6454	6.51	0.87	8.39E+06
**ST**	905.6262	ST t42:2	C48H91NO12S	[M-H]	904.6189	904.6228	4.31	0.88	9.56E+06
**ST**	907.6418	ST t42:1	C48H93NO12S	[M-H]	906.6346	906.6337	-0.99	0.88	4.44E+06
**ST**	915.6469	ST d44:3	C50H93NO11S	[M-H]	914.6396	914.6310	-9.40	0.87	3.45E+04
**ST**	917.6626	ST d44:2	C50H95NO11S	[M-H]	916.6553	916.6523	-3.27	0.87	2.31E+05
**ST**	921.6575	ST d43:1	C49H95NO12S	[M-H]	920.6502	920.6466	-3.91	0.88	1.41E+05
**ST**	931.6418	ST t44:3	C50H93NO12S	[M-H]	930.6346	930.6351	0.54	0.88	3.46E+04
**ST**	933.6575	ST t44:2	C50H95NO12S	[M-H]	932.6502	932.6474	-3.00	0.88	1.18E+05
**ST**	935.6731	ST t44:1	C50H97NO12S	[M-H]	934.6659	934.6570	-9.52	0.88	5.35E+04
**ST**	969.6059	diHexST d36:1	C48H91NO16S	[M-H]	968.5986	968.5978	-0.83	0.92	1.74E+05
**ST**	997.6372	diHexST d38:1	C50H95NO16S	[M-H]	996.6299	996.6240	-5.92	0.92	7.47E+04
**ST**	1023.6528	diHexST d40:2	C52H97NO16S	[M-H]	1022.6455	1022.6404	-4.99	0.90	7.14E+04
**ST**	1025.6685	diHexST d40:1	C52H99NO16S	[M-H]	1024.6612	1024.6565	-4.59	0.92	9.78E+04
**ST**	1039.6841	diHexST d41:1	C53H101NO16S	[M-H]	1038.6768	1038.6763	-0.48	0.92	4.12E+04
**ST**	1049.6685	diHexST d42:3	C54H99NO16S	[M-H]	1048.6612	1048.6550	-5.91	0.90	2.67E+04
**ST**	1051.6841	diHexST d42:2	C54H101NO16S	[M-H]	1050.6768	1050.6774	0.57	0.90	7.92E+05
**ST**	1053.6998	diHexST d42:1	C54H103NO16S	[M-H]	1052.6925	1052.6858	-6.36	0.92	3.41E+05

Table S2. Statistical analysis of data from GSL screening in aging rats and transgenic rat model for tau pathology. Data are shown as negative log (p-value of t-test).

Lipid class	Lipid species	Aging CN groups					Aging TG groups					TG vs. Controls					
		CN 4 / CN 6	CN 4 / CN 8	CN 4 / CN 10	CN 4 / CN 12	CN 4 / CN 14	TG 4 / TG 6	TG 4 / TG 8	TG 4 / TG 10	TG 4 / TG 12	TG 4 / TG 14	TG 4 / CN 4	TG 6 / CN 6	TG 8 / CN 8	TG 10 / CN 10	TG 12 / CN 12	TG 14 / CN 14
GANGLIOSIDES	GD0a 36:2	0.14	0.08	0.30	0.00	0.35	0.61	0.07	0.29	0.44	0.05	0.28	0.32	0.31	0.37	0.00	1.00
	GD0a 38:1	0.44	0.18	0.17	0.00	0.32	1.05	0.22	0.19	0.19	0.15	0.48	1.01	0.09	0.53	0.44	1.11
	GD1a d37:1	0.48	0.28	0.14	0.24	0.08	0.84	0.28	0.28	0.31	0.18	0.37	1.07	0.15	0.26	0.13	0.35
	GD1a d38:1	0.80	0.63	0.02	0.40	0.06	0.60	0.13	0.29	0.43	0.23	0.46	1.06	0.34	0.60	0.27	0.81
	GD1a d38:2	0.45	0.39	0.03	0.39	0.02	0.34	0.12	0.18	0.24	0.54	0.24	0.76	0.03	0.43	0.05	0.75
	GD1a d40:1	0.53	0.23	0.14	0.15	0.03	0.93	0.43	0.00	0.35	0.44	0.43	1.13	0.15	0.55	0.03	0.06
	GD1a-Ac d36:1	0.59	0.48	0.11	0.57	0.08	0.49	0.04	0.13	0.22	0.42	0.29	0.97	0.13	0.54	0.04	0.73
	GD1a-Ac d38:1	0.74	0.57	0.07	0.55	0.27	0.44	0.08	0.43	0.66	0.43	0.34	1.06	0.15	0.85	0.21	0.46
	GD1b 38:1	0.72	0.45	0.12	0.28	0.15	0.54	0.06	0.50	0.33	0.02	0.41	1.00	0.14	0.78	0.32	0.31
	GD1b-Ac d36:1	0.90	0.51	0.11	0.75	0.84	0.48	0.16	0.53	0.41	0.39	0.45	1.03	0.05	0.77	0.00	0.04
	GD1b-Ac d38:1	0.93	0.60	0.08	0.54	0.79	0.46	0.08	0.59	0.74	0.31	0.43	1.12	0.12	0.83	0.28	0.02
	GD2 d36:1	1.12	0.75	0.21	0.82	0.43	0.30	0.24	0.47	0.90	0.70	0.64	0.96	0.06	0.74	0.38	0.67
	GD3 d34:1	0.94	0.49	0.20	0.63	0.81	0.13	0.00	0.06	0.01	0.18	0.20	1.41	0.28	0.07	0.42	0.40
	GD3 d36:1	1.14	0.64	0.15	0.97	0.74	0.13	0.22	0.80	0.80	0.50	0.19	1.44	0.18	0.79	0.15	0.07
	GD3 d36:2	0.41	0.15	0.03	0.25	0.05	0.16	0.08	0.28	0.09	0.06	0.11	0.71	0.04	0.48	0.06	0.12
	GD3 d40:1	0.49	0.11	0.02	0.29	0.33	0.43	0.05	0.49	0.06	0.14	0.23	1.07	0.07	0.82	0.13	0.27
	GD3 d44:6	0.05	0.29	0.78	0.86	0.94	0.93	0.91	0.13	1.38	1.81	0.21	0.52	0.14	0.99	0.49	0.21
	GD3-Ac d36:1	0.51	0.17	0.15	0.63	1.05	0.54	0.16	0.31	0.02	0.01	0.68	0.42	0.45	0.50	0.09	0.42
	GD3-Ac d38:1	0.55	0.17	0.08	0.71	1.21	0.53	0.10	0.49	0.07	0.09	0.63	0.48	0.34	0.76	0.13	0.71
	GM1a d34:1	0.38	0.03	0.58	0.21	0.22	1.36	0.86	0.09	0.64	0.63	0.32	1.28	0.12	0.99	0.21	0.17
	GM1a d35:1	0.32	0.10	0.78	0.53	0.55	1.43	1.53	0.51	1.51	1.64	0.31	1.31	0.29	0.78	0.16	0.01
	GM1a d36:2	0.45	0.05	0.27	0.06	0.18	0.38	0.14	0.14	0.31	0.08	0.28	0.72	0.10	0.92	0.13	0.63
	GM1a d37:1	0.35	0.12	0.79	0.73	0.77	1.21	1.52	0.33	1.84	1.78	0.43	1.06	0.15	1.12	0.40	0.19
	GM1a d38:1	0.25	0.14	0.82	0.95	0.98	1.18	1.54	0.05	1.43	1.67	0.42	0.74	0.08	1.14	0.76	0.43

	GM1a d40:2	0.03	0.74	1.79	1.87	1.10	2.03	1.56	1.30	3.06	2.71	0.24	1.21	0.22	1.12	0.53	0.10
	GM1a d42:2	0.17	1.16	3.07	3.31	2.01	3.42	4.19	1.94	5.63	6.46	0.41	1.13	0.01	1.02	0.65	0.06
	GM1a t38:1	0.33	0.04	0.12	0.14	0.46	0.25	0.12	0.39	0.08	0.42	0.23	0.43	0.38	0.92	0.20	0.08
	GM1a t38:2	0.44	0.04	0.49	0.35	0.45	1.07	0.64	0.07	0.86	0.80	0.38	1.31	0.05	1.13	0.49	0.41
	GM1a t38:4	0.30	0.07	0.56	0.56	0.67	1.27	1.46	0.46	1.55	1.62	0.44	0.92	0.10	0.94	0.49	0.37
	GM2 36:2	0.46	0.08	0.19	0.14	0.09	0.45	0.28	0.27	0.53	0.30	0.43	0.54	0.13	0.58	0.28	0.49
	GM2 d34:1	0.22	0.04	0.51	0.33	0.18	1.33	0.87	0.59	1.40	1.29	0.37	1.16	0.04	0.49	0.01	0.16
	GM2 d38:1	0.38	0.01	0.43	0.50	0.46	0.85	1.22	0.27	1.44	1.57	0.48	0.66	0.12	0.78	0.46	0.18
	GM3 d34:1	0.91	0.75	0.02	0.46	0.62	0.67	0.41	0.18	1.29	0.52	0.29	1.94	1.03	0.01	1.52	1.04
	GM3 d36:1	0.95	0.56	0.03	0.47	0.44	0.31	0.25	0.32	0.01	0.10	0.13	1.63	0.84	0.41	0.40	0.49
	GM3 d38:2	0.45	0.32	0.05	0.44	0.38	0.07	0.18	0.01	1.30	0.03	0.18	0.43	0.34	0.16	1.66	0.27
	GM3 d40:1	0.48	0.31	0.09	0.12	0.08	0.66	1.29	0.35	2.56	1.37	0.39	0.69	0.96	0.01	1.75	0.58
	GM3 d42:2	0.52	0.31	0.25	0.00	0.16	1.39	2.04	0.79	3.35	2.19	0.22	2.16	2.02	0.28	3.19	1.83
	GM3 d42:3	0.22	0.00	0.04	0.26	0.18	0.76	0.46	0.56	1.83	0.26	0.26	0.63	0.07	0.19	1.69	0.13
	GM3 t38:1	0.33	0.03	0.07	0.01	0.04	0.48	0.18	0.49	1.69	0.82	0.28	0.53	0.20	0.07	0.68	0.35
	GM3 t40:1	0.15	0.00	0.29	0.11	0.03	0.75	1.42	0.55	2.22	1.59	0.21	0.57	0.67	0.06	1.29	1.06
	GM3 t42:3	0.21	0.15	0.41	0.19	0.18	1.40	1.69	1.81	4.44	3.27	0.45	0.81	0.15	0.19	1.56	1.01
	GT1a-Ac 36:1	0.55	0.20	0.06	0.61	0.10	0.39	0.29	0.56	0.36	0.55	0.17	1.22	0.27	0.84	0.09	0.59
	GT1a-Ac 38:1	0.11	0.14	0.20	0.10	0.05	0.01	0.48	0.42	0.07	0.21	0.04	0.11	0.76	0.66	0.03	0.02
	GT1a-Ac d36:1	0.63	0.37	0.01	0.71	0.23	0.20	0.28	0.53	0.48	0.56	0.21	0.86	0.10	0.75	0.04	0.39
	GT1a-Ac d38:1	0.76	0.48	0.00	0.66	0.70	0.07	0.28	0.85	0.81	0.54	0.26	0.73	0.06	1.12	0.20	0.12
	GT1b 35:1	0.82	0.44	0.59	0.94	0.25	0.17	0.44	0.05	0.54	0.47	0.31	0.81	0.19	0.12	0.12	0.25
	GT1b 37:1	0.61	0.28	0.15	0.37	0.15	0.87	0.01	0.15	0.14	0.19	0.38	1.39	0.08	0.03	0.16	0.02
	GT1b 38:1	1.14	0.85	0.47	0.79	0.64	0.50	0.37	0.40	0.65	0.52	0.61	1.20	0.01	0.45	0.11	0.39
	GT1b 38:2	0.83	0.68	0.51	1.19	0.66	0.50	0.70	0.28	0.44	0.61	0.46	1.07	0.38	0.22	0.23	0.19
	GT1b 40:1	0.78	0.34	0.30	0.44	0.31	0.71	0.24	0.13	0.00	0.01	0.57	1.13	0.31	0.28	0.05	0.23
	GT1b 40:4	0.97	0.62	0.40	0.61	0.59	0.47	0.34	0.12	0.33	0.38	0.62	1.08	0.24	0.26	0.18	0.38
	GT1b-Ac 36:1	0.91	0.62	0.21	1.03	0.60	0.46	0.39	0.35	0.49	0.50	0.39	1.14	0.09	0.43	0.19	0.17
	GT1b-Ac 38:1	0.98	0.77	0.25	0.94	1.24	0.39	0.42	0.37	0.74	0.58	0.51	1.07	0.08	0.53	0.08	0.03
	GT1b-Ac d38:1	1.12	0.83	0.26	0.99	1.33	0.36	0.37	0.45	0.87	0.64	0.52	1.07	0.03	0.56	0.05	0.03
	GT1b-Ac d38:2	0.69	0.45	0.20	0.76	0.87	0.34	0.46	0.39	0.29	0.47	0.33	0.93	0.29	0.47	0.11	0.04

	GT1b-Ac d40:1	0.88	0.54	0.12	0.58	0.77	0.67	0.13	0.25	0.23	0.11	0.57	1.13	0.07	0.61	0.06	0.02
	GT1b-Ac d40:4	0.92	0.81	0.32	0.85	1.20	0.33	0.48	0.42	0.82	0.72	0.49	0.94	0.05	0.52	0.11	0.02
	GT1b-Ac2 d36:1	0.63	0.37	0.03	0.76	0.94	0.32	0.20	0.34	0.35	0.48	0.40	0.57	0.23	0.66	0.01	0.08
	GT1b-Ac2 d38:1	0.88	0.70	0.10	0.96	1.47	0.28	0.31	0.53	0.83	0.72	0.38	0.84	0.00	0.69	0.04	0.27
	GT1b-Ac2 d38:3	0.58	0.45	0.18	0.63	0.94	0.25	0.30	0.36	0.27	0.59	0.47	0.39	0.33	0.65	0.07	0.10
	GT1b-Ac2 d40:1	0.81	0.56	0.02	0.68	1.31	0.39	0.15	0.40	0.55	0.33	0.46	0.83	0.04	0.85	0.16	0.32
	GT1b-Ac2 d40:4	0.80	0.84	0.27	0.87	1.46	0.19	0.36	0.56	0.75	0.96	0.46	0.60	0.02	0.67	0.08	0.17
	GT3-Ac 38:1	0.51	0.11	0.01	0.32	0.27	0.46	0.38	0.30	0.01	0.03	0.49	0.56	0.29	0.53	0.46	0.46
SULFATIDES	diHexST d36:1	1.32	1.21	1.50	2.29	2.98	0.14	1.13	0.87	1.03	2.53	0.39	0.91	0.08	0.00	0.94	0.69
	diHexST d38:1	0.85	0.58	0.98	1.55	2.28	0.23	0.25	0.26	0.70	1.95	0.67	0.43	0.27	0.03	0.17	0.21
	diHexST d40:1	0.74	0.52	0.91	1.33	1.89	0.42	0.30	0.32	0.73	1.62	0.60	0.59	0.36	0.12	0.00	0.02
	diHexST d40:2	0.45	0.28	1.00	1.31	2.64	0.36	0.90	1.13	0.92	2.96	0.53	0.28	0.98	0.46	0.01	0.19
	diHexST d42:1	0.68	0.38	0.92	1.08	1.91	0.37	0.15	0.36	0.07	1.33	0.56	0.50	0.30	0.15	0.38	0.17
	diHexST d42:3	0.69	0.35	1.54	1.68	2.68	0.06	2.38	1.01	1.48	4.30	0.33	0.41	1.63	0.27	0.40	0.01
	ST d34:1	1.18	1.25	1.10	1.84	2.48	0.34	1.50	1.29	1.90	3.11	0.27	0.67	0.21	0.27	0.14	0.32
	ST d34:2	0.41	0.06	0.45	0.91	0.96	0.22	0.65	0.32	0.08	0.62	0.15	0.04	1.22	0.05	0.92	0.19
	ST d36:1	1.19	1.39	1.08	1.94	2.17	0.36	1.14	1.24	1.65	2.69	0.34	0.47	0.09	0.37	0.21	0.46
	ST d36:2	0.99	0.63	0.88	1.43	1.75	0.46	1.33	1.42	0.95	1.96	0.15	0.45	0.49	0.33	0.43	0.46
	ST d38:1	0.39	0.21	0.06	0.37	0.92	0.43	0.03	0.08	0.08	0.39	0.60	0.16	0.37	0.47	0.10	0.28
	ST d38:2	0.30	0.04	0.01	0.19	0.73	0.22	0.17	0.23	0.10	0.58	0.38	0.12	0.77	0.54	0.07	0.17
	ST d40:1	0.58	0.53	0.46	1.01	1.42	0.23	0.46	0.32	0.30	1.47	0.63	0.18	0.57	0.33	0.05	0.08
	ST d40:2	0.23	0.05	0.01	0.13	0.78	0.47	0.10	0.26	0.18	0.71	0.56	0.05	0.75	0.79	0.28	0.14
	ST d41:2	0.03	1.02	1.08	1.20	0.66	0.85	1.17	1.12	3.96	3.63	0.39	0.10	0.77	0.47	0.40	0.29
	ST d42:1	0.76	1.11	2.27	2.73	2.80	0.02	0.84	0.17	1.29	1.99	0.72	0.10	0.34	0.51	0.23	0.27
	ST d42:2	0.18	0.14	0.15	0.01	0.45	0.20	0.05	0.05	1.03	0.03	0.31	0.06	0.78	0.54	0.38	0.26
	ST d42:3	0.33	0.02	0.37	0.83	1.18	0.08	0.75	0.90	0.15	1.41	0.20	0.05	1.21	0.47	0.37	0.01
	ST d43:1	0.06	1.02	1.20	1.30	2.02	1.26	1.37	1.66	3.56	4.38	0.40	0.32	0.56	0.06	0.42	0.14
	ST d44:2	0.02	0.03	0.00	0.49	0.93	0.40	0.44	0.25	0.06	0.62	0.13	0.22	0.86	0.09	0.43	0.05
	ST t36:1	0.96	1.57	2.13	3.02	3.47	1.31	2.94	2.58	3.93	5.39	0.15	0.01	0.60	0.17	0.42	0.02
	ST t38:0	0.48	0.17	0.63	1.24	0.91	0.35	0.64	0.81	0.60	1.45	0.39	0.16	0.75	0.35	0.20	0.49
	ST t40:1	0.24	0.05	0.01	0.09	0.21	0.94	0.28	0.23	0.47	0.46	0.57	0.35	0.23	0.20	0.11	0.00

	ST t42:1	0.25	0.20	0.10	0.22	0.07	0.75	0.12	0.39	0.14	0.27	0.51	0.33	0.07	0.03	0.09	0.25
	ST t42:2	0.20	1.42	1.33	1.67	1.14	1.56	2.15	0.81	4.72	3.92	0.47	0.26	0.55	0.53	0.28	0.21
	ST t44:2	0.06	0.30	0.12	0.14	0.13	0.80	0.03	0.59	0.69	0.17	0.37	0.32	1.31	0.01	0.33	0.15

Table S3. Results from reproducibility testing.

Analyte	Average Peak Area (n=12)	RSD (%)
GD1a d36:1	1.6E+08	15
GD1b 36:1	3.5E+07	18
GM1a d36:1	7.1E+06	3
GM3 d36:1	8.2E+06	15
ST 36:1	1.1E+08	10

References:

1. Hájek, R.; Jirásko, R.; Lísa, M.; Cífková, E.; Holčapek, M.; Haek, R.; Jirako, R.; Lísa, M.; Cífkova, E.; Holcˇ, M.; et al. Hydrophilic Interaction Liquid Chromatography-Mass Spectrometry Characterization of Gangliosides in Biological Samples. *Analytical Chemistry* **2017**, *89*, 12425–12432, doi:10.1021/acs.analchem.7b03523.
